# Luck, jobs and learning

**DOI:** 10.7554/eLife.00676

**Published:** 2013-04-16

**Authors:** Eve Marder

**Affiliations:** Department of Biology and the Volen National Center for Complex Systems, Brandeis University, Waltham, United Statesmarder@brandeis.edu

**Keywords:** Living science, careers in science, grad school, postdoc

## Abstract

**Eve Marder** believes that many of the most important events in our lives, both personal and professional, depend to some degree on luck or chance.

I learned to read while living in a town that was so small it didn't have a high school, but it did have a public library, with a tiny room devoted to children's books. The books were arranged by school year; for some reason, I read all of the books intended for first graders before starting on those intended for second graders, thus marching from fairy tales to history to science. When I was about seven, I was reading a science book, and a visiting relative asked me the inevitable question: ‘What do you want to be when you grow up?’ When I answered, ‘A scientist’, I received smiles and nods of approval, quickly teaching me that this was an acceptable answer that made annoying adults leave me in peace. Consequently, this became my stock answer whenever I was asked what I wanted to be when I grew up, even though I did not know the first thing about what being a scientist meant.

I started college (university for non-Americans) in 1965; the United States was in the throes of the civil rights movement, and I intended to be a civil rights lawyer or an investigative journalist. A series of accidents (luck?) led me to change my major from politics to biology, and I started graduate school in biology rather than going to law school. Of course, even then I was clueless about what it would mean to be a scientist or, for that matter, a lawyer or a journalist. But I became a scientist. Today, 39 years after my PhD, I spend most of my time talking to people, reading and writing—much as I might have done as a lawyer or journalist.

Our present world is filled with great angst. Our junior faculty are writing too many grant applications for not enough money. Our postdocs rightfully feel that they are in purgatory, not knowing when and if there will be an academic position for them, should they desire one. Our graduate students are watching the struggles of postdocs and faculty. For me, this era is especially frustrating, because it is a time of extraordinary opportunity for scientific discovery, and it is criminal that our young scientists can not experience the excitement and challenge of scientific discovery without being worried about their futures.

All of us should also be aware that we have the potential to be successful in careers other than academic science.

There is no right answer to the question of how long a talented scientist can or should remain in a ‘looking for a job’ limbo. Every individual must take into account their own ambitions and circumstances as they try to answer this question. And all of us should also be aware that we have the potential to be successful in many careers, in and out of science. (I sometimes fantasize that had I become a lawyer I could have become a Supreme Court Justice, and had far greater influence on the course of history than my career in science will ever achieve.)

It is important to recognize that some of the most important events in our lives, as scientists and people, depend on luck or chance. We accept that the randomness of the world plays an important role in finding a spouse, significant other, and friends. I met my husband on a hot August day when we were both outside the student center at Brandeis University—my newspaper blew away and he rescued it. Perhaps had that not been a windy day, or perhaps if he had been 10 minutes later, we might never have talked? Calling this luck does not devalue its importance, or the significance he has in my life.

**Figure 1:**
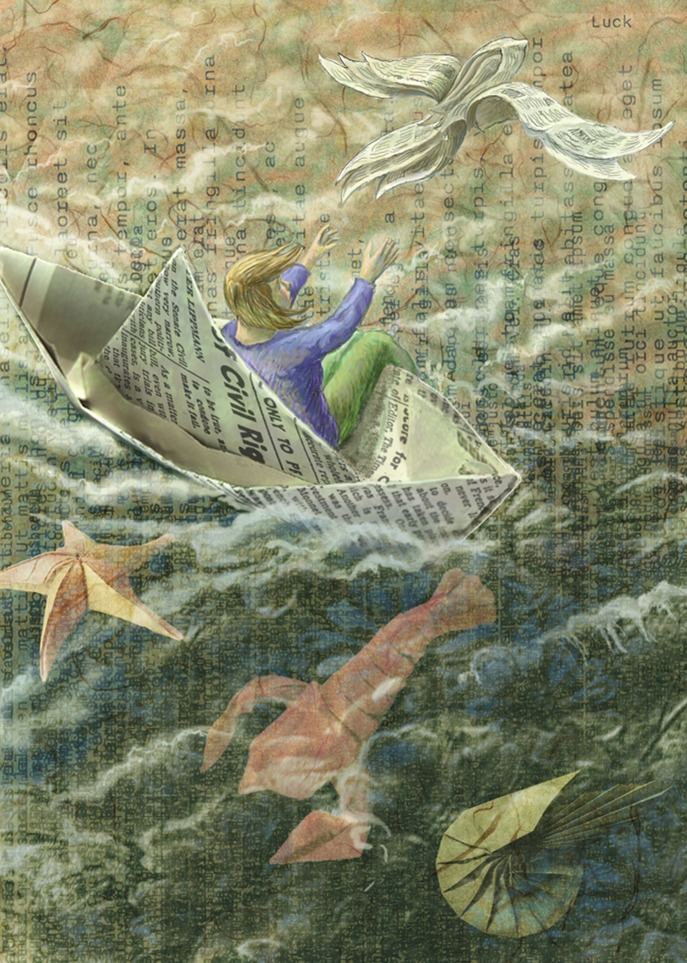
Some of the most important events in our lives, both as scientists and people, depend on luck. Chance events played a large role in Eve Marder meeting her husband and becoming a scientist rather than a journalist or lawyer.

ILLUSTRATION: BEN MARDER

As scientists, we hope that our papers and grant applications are evaluated objectively on their intrinsic value. Nonetheless, there is a great deal of luck involved: for example, the fate of our papers or grant applications depends on who agrees to review them, and on how their personal perspectives influence the review. Indeed, their fate might depend on whether an editor or reviewer is in a good or bad mood, and on whether he or she has the requisite patience and forgiveness to deal with a poorly written paragraph or anything that is difficult to understand. Of course we all try to be fair as reviewers, but if we are honest with ourselves we know that each of us is more objective some days than others. And as editors we try our best to be wise, but we are aware that the final outcome of a paper depends also on who agrees to referee it.

There is even more luck involved in the search for a postdoc or faculty position.

There is even more luck involved in the search for a postdoc or faculty position. Luck plays a role in whether you approach a potential postdoctoral supervisor after he or she has just received a new grant and has space, or has just accepted three new postdocs and has a full lab. Yes, your accomplishments as a graduate student will be important when you are looking for a good postdoc position, but timing and chance also matter as you negotiate for a specific position.

In the search for a faculty position, timing and luck play an even larger role. A strong record of accomplishment as a postdoc is necessary, but may not be sufficient. Yes, good ideas for future work are essential. But luck determines which departments have openings when you are ‘on the market’. Luck governs the ‘fit’ between an individual and a specific department, and it is foolishness for anyone involved (including the faculty doing the search) to assume that a search committee can know the future or has the wisdom to spot the ‘best’ candidate. Whether or not they are willing to acknowledge it, search committees are always guessing as they look for a new colleague. Some search committees will follow their gut instincts and respond to a sense that they have identified a special talent in a candidate. Other search committees will default by responding to proxies for excellence, such as where a paper was published. Others will do due diligence and talk to as many people they can find who know their top candidates. Other search committees do a complex mixture of all of the above. But, at the end of the day, the offer depends on both the demonstrated talent of the candidate and good luck.

Each year, there are more outstanding candidates for faculty positions than there are positions. I know young people who found the job of their dreams after three or more years of waiting and searching. And I know a number of superb young scientists, who took their failure to find a position in a given year not as a personal failure, but as a signal from the heavens that they might look to other career options. Many of our strongest early scientists are deciding to leave academic science, with a better understanding of themselves and their talents than they had when they started graduate school, and embark on careers that will draw on their skills and scientific training.

Although getting a job and a grant was probably easier 30 or 40 years ago (if you weren't female or minority), most of my graduate school cohort are not in academic science today. Instead, they are in industry, secondary education, journalism, medicine, business and law. Looking back, it is clear that for many, their dreams changed, as they took advantage of opportunities and challenges unanticipated when they started graduate school. Time spent as a graduate student or as a postdoc is never wasted, regardless of your eventual career path. In all that we do in life, in academic science or other arenas, the most important attribute is the ability to continue learning. Science and the rest of the world changes around us. It is the ability to learn that enables a graduate student or a postdoc to be successful in careers other than scientific research. Similarly, the academic scientists who contribute the most are those who can create new knowledge precisely because they continue to learn throughout their lives.

